# Effects of Additional Ethanol Exposure and Post-Curing Time on Color Stability, Water Sorption, and Solubility of 3D-Printed Permanent Restorative Resins

**DOI:** 10.3390/jfb17100510

**Published:** 2026-10-11

**Authors:** Merve Güngör, Abdurrahman Yalçın, Hatice Ortaç, Buket Ayna

**Affiliations:** 1 Department of Pedodontics, Faculty of Dentistry, Dicle University, 21280 Diyarbakır, Türkiye; 2 Department of Restorative Dentistry, Faculty of Dentistry, Dicle University, 21280 Diyarbakır, Türkiye; 3 Department of Biostatistics, Faculty of Medicine, Dicle University, 21280 Diyarbakır, Türkiye

**Keywords:** 3D printing, dental biomaterials, post-processing, color stability, water sorption and solubility

## Abstract

Background: The aim of this study was to evaluate the effects of additional ethanol exposure and additional post-curing times, applied following the standard post-printing protocols recommended by the manufacturer, on the color stability, water sorption, and solubility of two 3D-printed permanent restorative resins. Methods: A total of 160 disk specimens were prepared using Crowntec and VarseoSmile TriniQ. For each material, two independent experimental arms were established: additional ethanol exposure and additional post-curing. Within each arm, separate specimens were assigned to 0 (control), 3, 10, or 30 min conditions following completion of the manufacturer-recommended standard post-processing protocol. Water sorption (Wsp) and solubility (Wsl) were evaluated first using a gravimetric approach, after which the same conditioned specimens were subjected to color evaluation using the CIEDE2000 (ΔE_00_) system during a 28-day continuous black tea immersion challenge. Results: Additional ethanol exposure resulted in lower ΔE_00_ values on Day 1 compared to the control groups for both materials; however, this difference did not persist over time. In Crowntec, 30 min of additional post-curing was associated with lower Wsp than the 3- and 10-min conditions and with lower ΔE_00_ at later color-evaluation time points. In VarseoSmile TriniQ, 30 min of additional ethanol exposure resulted in higher Wsp than the 10-min condition. In VarseoSmile TriniQ, however, extended post-curing did not provide a similar benefit. Additional processing durations did not significantly alter Wsl within either material in the independent-group analyses; however, factorial analysis identified a significant material × duration interaction in the post-curing arm, and VarseoSmile TriniQ exhibited higher Wsl values than Crowntec under their respective manufacturer-specific processing workflows. Conclusions: Extending post-processing beyond manufacturer-validated protocols did not consistently improve material performance and, under some conditions, was associated with less favorable outcomes. Accordingly, the additional ethanol exposures investigated here should be regarded as experimental over-processing challenges rather than as alternative clinically recommended washing protocols.

## 1. Introduction

3D printing technologies are becoming increasingly widespread in restorative dentistry due to their advantages in controlled digital production, the ability to create complex geometries, and low material waste [1,2]. Advances in resin formulations have enabled the use of 3D printing not only for temporary restorations but also for permanent restorative materials [1]. Current systematic evidence indicates that certain properties of 3D-printed long-term restorative resins are less predictable compared to those of conventional materials; therefore, the optimization of material and post-processing protocols is crucial [1,2].

In photopolymer-based 3D printing, the production process continues with cleaning and post-curing procedures performed after the print is completed. While the cleaning process aims to remove unpolymerized resin residues from the surface, the post-curing process contributes to the progression of polymerization and the development of the material’s final properties [2]. It has been reported that variables such as the solvent used, the cleaning method and duration, as well as the post-curing duration, temperature, light conditions, and equipment used, can affect the material’s mechanical, physical, surface, and optical properties [2,3,4,5].

Jin et al. reported that while an extended washing time in 3D-printed crown-bridge resins reduces the amount of residual monomer, it can lead to a decrease in the degree of conversion and certain mechanical properties beyond a certain duration [6]. Similarly, it has been shown that different post-rinse solutions used in 3D-printed composite resins developed for permanent restorations can affect water sorption, solubility, and various physical properties [7]. A recent study also reported that extended washing times affect color stability differently depending on the material [8]. These findings indicate that the type of solvent and contact time must be considered not only in terms of surface cleaning but also in terms of the material’s subsequent performance.

The post-curing time is also an important post-processing variable that affects the final properties of 3D-printed resins; it has been reported that different post-curing times can affect the degree of conversion, hardness, mechanical properties, color stability, surface properties, water sorption, and solubility of 3D-printed resins [3,4,5]. A recent study reported that an extended post-curing time increases the degree of conversion and microhardness while reducing the tendency toward discoloration; however, the effect is also influenced by the device used [3]. Therefore, the effect of post-curing time depends not only on the duration of application but also on the material used and the post-curing conditions.

In addition to optical properties, water sorption (Wsp) and solubility (Wsl) are important parameters for evaluating the behavior of resin-based restorative materials in an aqueous environment. Current data indicate that these properties are influenced by material-specific factors, such as the resin matrix, filler system, and manufacturing method, and may vary among different materials [8]. Furthermore, the fact that post-rinse solutions [7] and post-curing time [5] can affect the Wsp and Wsl values supports the relationship between post-processing conditions and hydrolytic behavior.

Existing studies have demonstrated the effects of different total washing times or different post-curing times on material properties [3,4,5,6,8]. However, the effects of additional solvent exposure or additional post-curing time applied after completion of the manufacturer-recommended material-specific standard post-processing protocol on color stability, water sorption, and solubility have not been sufficiently clarified. Comparing the responses of different permanent 3D-printed resins to additional post-processing steps is important for understanding the material-specific effects of these additional treatments.

Therefore, the aim of this study is to evaluate, in two separate experimental groups, the effects of different additional ethanol exposure times and additional post-curing durations on the color stability, water sorption, and solubility of VarseoSmile TriniQ and Crowntec 3D-printed permanent restorative resins. Additional ethanol exposure and additional post-curing applications were evaluated independently of one another.

The null hypotheses of the study were formulated as follows: H_01_: Different additional ethanol exposure times applied following the material-specific standard cleaning protocol have no significant effect on the color change, water sorption, and solubility values of the 3D-printed permanent restorative resins under investigation; H_02_: Different additional post-curing durations applied following the standard post-curing procedure recommended by the manufacturer have no significant effect on these properties; H_03_: There is no significant difference between VarseoSmile TriniQ (VT) and Crowntec (CT) in terms of the evaluated properties of color change, water sorption, and solubility.

## 2. Materials and Methods

### 2.1. Study Design

This randomized in vitro study was conducted to evaluate the effects of additional ethanol exposure and additional post-curing time—applied following the standard post-printing protocols recommended by the manufacturer—on the color stability, water sorption, and solubility of 3D-printed permanent restorative resins. The study protocol was approved by the Ethics Committee of the Faculty of Dentistry, Dicle University (decision no.: 2026-40, protocol date: 24 June 2026).

**Figure 1:**
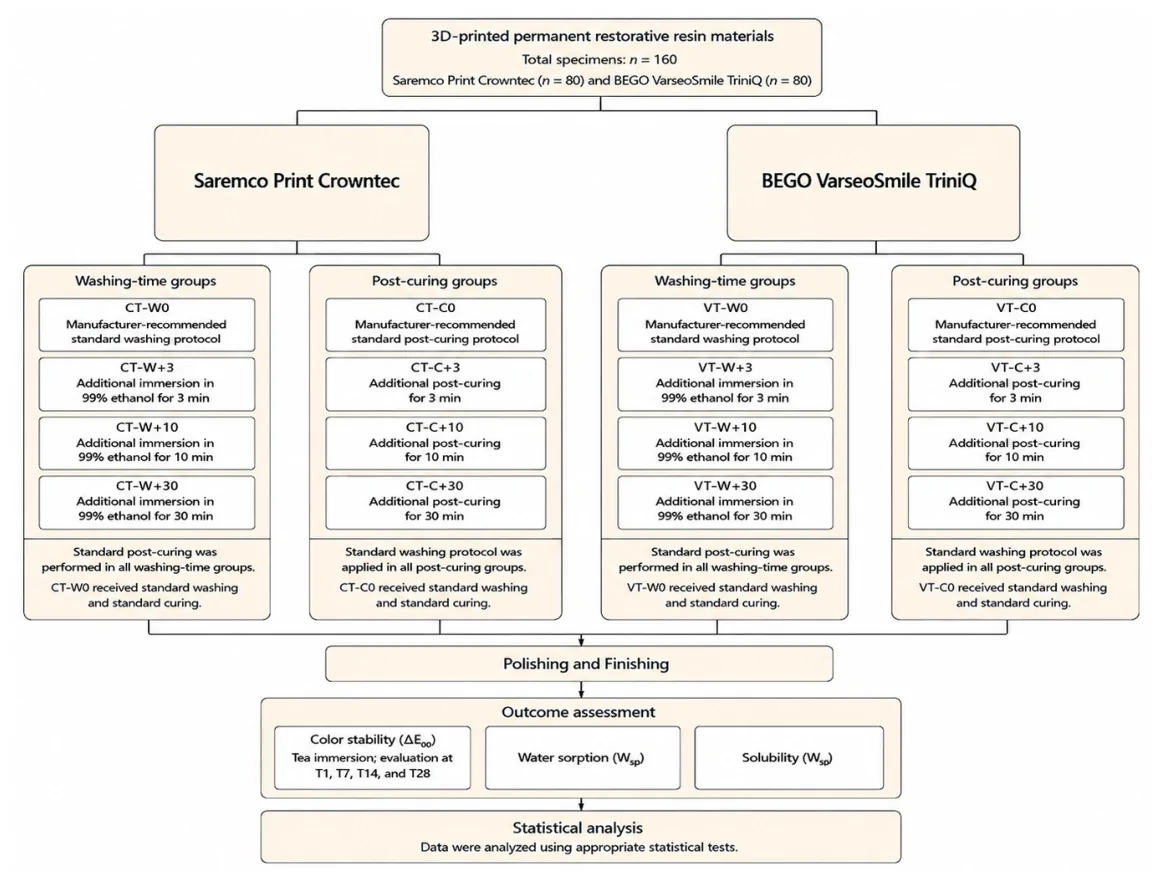
Flowchart illustrating the study’s experimental design, group allocation, and post-printing treatment protocols.

In the study, two post-printing treatment variables were evaluated in independent experimental groups: (1) the duration of additional ethanol exposure following standard cleaning, and (2) the duration of additional post-curing following standard post-curing. In the groups where ethanol exposure was evaluated, the post-curing protocol was kept constant, while in the groups where post-curing duration was evaluated, the material-specific standard cleaning protocol was kept constant. Therefore, the combinations of the two treatment variables and the interactions between them were not evaluated. The experimental design of the study, group allocation, and the applied post-printing treatments are summarized in Figure 1.

### 2.2. Determination of Sample Size

The sample size for this study was determined using an a priori power analysis conducted with the G*Power 3.1 software (Heinrich-Heine-Universität Düsseldorf, Düsseldorf, Germany). This power analysis was performed to support the primary comparison of interest in the present study, namely the between-material comparison (SAREMCO vs. BEGO). The effect size was calculated based on data from Batmaz et al. [9], who evaluated the effect of post-printing washing protocols on similar 3D-printed permanent restorative resins. Cohen’s d = 1.60 was adopted for the comparison between the two independent groups. In a two-tailed test, α = 0.05, target power (1−β) = 0.90, and a 1:1 group ratio were specified. The analysis determined that a minimum of 10 samples were required for each subgroup. Accordingly, eight subgroups were formed for each material, and a total of 160 samples—10 samples per subgroup—were included in the study. This calculation should not be interpreted as indicating sufficient statistical power for all analyses performed in the present study; power for the secondary comparisons involving multiple treatment durations, repeated color measurements over time, and material × duration interaction effects was not separately calculated, and the results of these exploratory analyses should be interpreted with this limitation in mind.

The study utilized two different 3D printing resins developed for the production of permanent restorations. To minimize potential variability arising from color differences between the materials, the A2 shade was selected for both resins. Information on the manufacturers and compositions of the materials used in the study is presented in Table 1.

### 2.3. Design and Fabrication of Samples

Disc-shaped samples were designed using computer-aided design software (Exocad DentalCAD, 3.1; Exocad GmbH, Darmstadt, Germany) to be 8 mm in diameter and 2 mm thick. The specimens were fabricated using a DLP-based Asiga MAX UV 3D printer (Asiga, Sydney, Australia) with a wavelength of 385 nm at a layer thickness of 50 µm. The disks were positioned at a 0° print orientation, with their flat surfaces parallel to the build platform. This orientation was selected to standardize the fabrication conditions across all specimens and both materials, thereby minimizing orientation-related variability in the evaluated outcomes. The same orientation was maintained for all experimental groups, while material-specific validated printing parameters were applied. A total of four independent print batches were performed, with 45 specimens fabricated in each batch, resulting in 180 specimens. For each material, one print batch was assigned to the additional ethanol exposure arm and another to the additional post-curing arm. Thus, each print batch corresponded to one independent experimental arm comprising four treatment conditions. To prevent cross-contamination between materials, a separate resin tank was used for each material. Following printing, all specimens were visually inspected for surface defects, deformation, or dimensional distortion. Four specimens were excluded because of printing defects. From the remaining defect-free specimens, 40 specimens from each experimental arm were selected for inclusion, resulting in a total of 160 specimens.

### 2.4. Formation of Experimental Groups

For each material, two independent experimental arms were established: additional ethanol exposure and additional post-curing. Each experimental arm originated from an independent print batch. Following printing and initial specimen inspection, eligible specimens within each arm were allocated into four subgroups (*n* = 10) using computer-assisted permuted-block randomization with a block size of four, with one specimen assigned to each treatment condition within each block. Allocation was therefore performed after printing and specimen quality assessment. In the additional ethanol exposure arm, all groups subsequently underwent the same standard post-curing protocol, whereas in the additional post-curing arm, all groups underwent the same material-specific standard cleaning protocol. Separate W0 and C0 control groups were intentionally maintained because the additional ethanol exposure and additional post-curing experiments were conducted as independent experimental arms originating from separate print batches. Each 45-specimen print batch was randomized within its respective four-group experimental arm; therefore, each arm included its own standard-processing control. This design allowed the 3-, 10-, and 30-min experimental conditions to be compared with a control specimen set originating from the same print batch and manufacturing workflow, rather than with a shared control produced in a separate build. The distribution of samples among the experimental groups and the applied post-printing treatment protocols are provided in Supplementary Table S1.

### 2.5. Post-Printing Cleaning and Additional Ethanol Exposure

After the printing process was completed, all samples were cleaned according to the standard cleaning protocol recommended by the manufacturer for the specific material.

VarseoSmile TriniQ specimens were subjected to a 3-min pre-cleaning in an ultrasonic bath with 96% ethanol, followed by a 2-min final cleaning in fresh 96% ethanol, in accordance with the manufacturer’s validated cleaning protocol. The specimens were then rinsed with fresh ethanol and completely dried using compressed air [10].

The Crowntec samples were cleaned using the manual cleaning protocol recommended by the manufacturer. In this study, uncured resin residues were removed using a cloth and brush soaked in 96% ethanol until the surface achieved a slightly matte appearance. The samples were not immersed in ethanol during the standard cleaning process and were thoroughly dried with compressed air after the procedure [11,12]. This procedure is consistent with the manufacturer’s recommendations for manual cleaning with 96% alcohol and avoiding alcohol immersion.

After completing the standard cleaning procedures, samples in the experimental groups were soaked in 99% ethanol for 3, 10, or 30 min to evaluate the effect of additional ethanol exposure. The additional immersion step was performed using 99% ethanol, whereas the manufacturer-recommended standard cleaning procedures involved 96% ethanol. Accordingly, this experimental arm was designed to evaluate different durations of additional 99% ethanol exposure following standard cleaning, rather than the isolated effect of extending the manufacturer-recommended washing time. No additional ethanol exposure was applied to the control groups. Following the additional ethanol exposure, the samples were thoroughly dried with compressed air, and the standard post-curing protocol recommended by the manufacturer for the specific material was applied to all groups, including the control groups. The additional 3-, 10-, and 30-min ethanol immersions were experimental excessive-solvent challenges conducted outside the manufacturer-validated processing workflows and should not be interpreted as alternative recommended cleaning protocols. This distinction is particularly important for Crowntec, for which the manufacturer recommends manual alcohol cleaning while avoiding alcohol immersion. Because the manufacturer-recommended standard cleaning protocols differed between the two materials, the additional ethanol exposure groups should not be interpreted as having equivalent total solvent exposure times. VarseoSmile TriniQ underwent approximately 5 min of ethanol immersion during its standard cleaning protocol before the additional exposure, whereas Crowntec was manually cleaned without ethanol immersion. Therefore, the +3, +10, and +30 min conditions represent equal additional exposure durations relative to each material’s own standard protocol rather than equivalent cumulative ethanol exposure between materials.

### 2.6. Standard and Additional Post-Curing Procedures

All specimens in the post-curing test group were first subjected to the material-specific, manufacturer-recommended standard cleaning protocol, and the specimens were thoroughly dried. All post-curing procedures were performed using the Asiga Cure (Asiga, Sydney, Australia) device.

For the VarseoSmile TriniQ samples, a dose-controlled post-curing program validated by the manufacturer for the Asiga Cure was applied. The program was run at a temperature of 25 °C, a target dose value of 17.5, a power level of 100%, and under vacuum conditions; the vacuum target value was set to 0.010, the minimum vacuum value to 0.012, and no gas was applied [10]. BEGO’s current compatibility document verifies these parameters for Asiga Cure and defines a target-dose-based program rather than a fixed post-curing time. Because this manufacturer-validated TriniQ program is dose-controlled rather than time-controlled, the standard cycle cannot be assumed to be directly equivalent to the time-based Crowntec protocol in terms of curing duration or total radiant exposure. Irradiance, wavelength range, actual delivered radiant energy, and the effective duration of the standard TriniQ cycle were not recorded as experimental output parameters in the present study. Crowntec specimens were subjected to the standard post-curing protocol validated by the manufacturer for the Asiga MAX UV–Asiga Cure combination. Accordingly, the samples were subjected to post-curing in the Asiga Cure unit for 10 min at 25 °C under vacuum [13]. Saremco’s current compatibility document reports that a 10-min post-curing protocol at 25 °C under vacuum has been validated for this combination.

After completing the material-specific standard post-curing procedures, time-controlled additional post-curing for 3, 10, or 30 min was applied to the experimental groups using the same Asiga Cure device to evaluate the effect of extended post-curing time; no additional post-curing was applied to the control groups. In the time-controlled applications, the temperature (25 °C) and vacuum conditions used in the standard protocols were maintained; in the TriniQ groups, the settings for 100% power level and no gas application were not altered, and the target dose value was not used as a criterion for terminating the additional treatment.

The additional 3-, 10-, and 30-min post-curing periods were experimental conditions applied after completion of each material’s manufacturer-validated standard curing procedure. Because the baseline TriniQ and Crowntec protocols were defined using different control approaches (dose-controlled versus time-controlled), identical additional curing durations should not be interpreted as producing identical total curing times or total radiant exposures between the two materials.

### 2.7. Finishing and Polishing Procedures

After all post-processing steps were completed, finishing and polishing procedures were performed by the same operator to ensure surface standardization of the specimens. The specimens were polished using the two-stage diamond-particle polishing system Clearfil Twist Dia (Kuraray Noritake Dental Inc.Tokyo, Japan) under water cooling at 10,000 rpm, with each polishing spiral applied for 30 s. Subsequently, Lucida diamond polishing paste (DiaShine, Lynnwood, WA, USA) was applied for 60 s at 5000 rpm using a felt polishing disc. All procedures were performed with continuous motion, and polishing pressure was manually controlled by the same operator but was not instrumentally standardized. Because specimen dimensions were not measured immediately before polishing, the exact amount of material removed during the finishing and polishing procedures could not be directly quantified. After polishing, the final dimensions of each specimen were measured using a digital caliper (Absolute Digimatic; Mitutoyo, Japan) with a precision of 0.01 mm. These actual post-polishing dimensions, rather than the nominal CAD dimensions, were used for the subsequent specimen-volume calculations. After the finishing and polishing processes were completed, water sorption (Wsp) and solubility (Wsl) evaluations were first conducted on all samples; following the completion of these analyses, the same samples were subjected to a color stability evaluation.

### 2.8. Evaluation of Water Sorption and Solubility

Water sorption (Wsp) and solubility (Wsl) were evaluated using an ISO 4049-based gravimetric approach adapted for the specimen dimensions used in the present study. Because these dimensions differed from those specified in ISO 4049, the procedure should not be regarded as fully ISO 4049-compliant. ISO 4049:2019 is the current standard for polymer-based restorative materials and covers water sorption and solubility evaluations. However, while the standard method specifies specimens with a diameter of 15.0 ± 0.1 mm and a thickness of 1.0 ± 0.1 mm, the literature reports the use of different specimen sizes, including 8 × 2 mm discs [14].

In this study, 8 mm diameter and 2 mm thick disk specimens were used to ensure standardization of specimen dimensions across all experimental evaluations. These dimensions have previously been used directly in the evaluation of water sorption and solubility of composite resins [15].

The specimens were initially placed in a desiccator and dried until a stable mass was obtained. However, the exact desiccation temperature, desiccant type, total drying duration, and a predefined numerical threshold for the maximum acceptable mass change between consecutive measurements were not prospectively recorded. The initial dry mass of each sample that reached a constant mass was measured using an analytical balance with a precision of 0.1 mg (AS 220 C2; Radwag, Radom, Poland) and recorded as m_1_. Determining the constant initial mass after desiccation is the fundamental procedure used in gravimetric Wsp/Wsl methods [16].

The actual post-polishing diameter and thickness of each specimen were used to calculate its individual volume (V) according to the disk geometry:

V = πr^2^h

Here, *r* represents the sample’s radius (mm), and *h* represents its thickness (mm).

After the initial constant masses were determined, the samples were left in distilled water at 37 °C for 7 days. Following the seven-day water exposure, the samples were removed from the water, excess water on their surfaces was gently blotted away with absorbent paper, and they were weighed on an analytical balance. The mass obtained after water exposure was recorded as m_2_. A seven-day water exposure at 37 °C is a condition commonly used in Wsp/Wsl protocols based on ISO 4049 [17].

Following the m_2_ measurement, the specimens were returned to the desiccator and re-dried until mass stabilization was achieved using the same laboratory procedure applied for the initial drying step. The final stabilized dry mass obtained after re-drying was recorded as m_3_. This gravimetric approach, which utilizes the initial dry mass (m_1_), the mass after water exposure (m_2_), and the mass after re-drying (m_3_), has also been applied to 3D-printed permanent restorative resins [16].

Water sorption and solubility values were calculated using the following equations:

Wsp = (m_2_ − m_3_)/V

Wsl = (m_1_ − m_3_)/V

Here, Wsp represents water sorption (µg/mm^3^), Wsl represents solubility (µg/mm^3^), m_1_ represents the initial constant dry mass (µg), m_2_ represents the mass after water exposure (µg), m_3_ represents the constant dry mass after re-drying (µg), and V represents the sample volume (mm^3^). These formulas have been used in gravimetric evaluations based on ISO 4049 and in recent studies examining 3D-printed permanent restorative resins, including Crowntec [18].

All weighings were performed under standardized laboratory conditions using the same analytical balance, and the Wsp and Wsl values were expressed in µg/mm^3^. The analytical balance had a readability of 0.1 mg. Therefore, very small mass differences, particularly those used for the calculation of Wsl, should be interpreted with consideration of the analytical resolution of the balance. No chemical analysis of the storage medium was performed; therefore, Wsl was interpreted only as a gravimetric solubility measure and not as a measure of the identity or concentration of specific released compounds.

### 2.9. Evaluation of Color Stability

Color measurements of the samples were performed on a standardized white background using a VITA Easyshade V spectrophotometer (VITA Zahnfabrik, Bad Säckingen, Germany). The device was calibrated prior to each measurement in accordance with the manufacturer’s recommendations. The samples were placed on a white background with the measurement surface facing upward, and the measurement probe was positioned perpendicular to the surface at the center of the sample. Three consecutive measurements were taken for each sample, and the average of the obtained values was used in statistical analyses. Similarly, the use of three repeated measurements on a white background with the VITA Easyshade V and the use of average L*, a*, and b* values has been applied in previous dental color studies [19].

Color coordinates were recorded according to the Commission Internationale de l’Éclairage (CIE) Lab* color system. The L* coordinate represents lightness, the a* coordinate represents the green–red axis, and the b* coordinate represents the blue–yellow axis. Following completion of the water sorption and solubility procedures, including water immersion and re-desiccation, the same specimens were used for color evaluation. The baseline color measurement for the subsequent staining experiment was then recorded as T0. Thus, T0 represented the baseline color of previously conditioned specimens rather than that of specimens measured immediately after fabrication and polishing.

The color change occurring in the samples was calculated using the CIEDE2000 color-difference formula (ΔE_00_). In the CIEDE2000 calculation, the parametric factors KL, KC, and KH were assumed to be 1 for standard conditions [20]. The 50:50 perceptibility and acceptability thresholds reported by Paravina et al. were used in the clinical interpretation of the ΔE_00_ values. Accordingly, ΔE_00_ = 0.8 was accepted as the perceptibility threshold (PT) and ΔE_00_ = 1.8 as the acceptability threshold (AT) [21].

Black tea solution (Lipton Yellow Label; Unilever) was used as the coloring medium in the evaluation of color stability. The tea solution was prepared by steeping one tea bag in 500 mL of boiled distilled water for 15 min. After the solution reached room temperature, the samples were fully immersed and incubated at 37 °C. The tea solution was prepared fresh and replaced every 24 h to minimize changes in concentration. Exposure to the chromogenic solution at 37 °C and a daily solution replacement protocol have previously been used for permanent 3D-printed restorative resins [22]. The continuous immersion protocol used in the present study was intended as a severe accelerated staining challenge for comparative evaluation and was not designed to reproduce the intermittent beverage exposure that occurs under normal clinical conditions.

Color measurements were taken at the start (T0) and then repeated on days 1 (T1), 7 (T7), 14 (T14), and 28 (T28) after the samples were left to stand in the tea solution. Before each measurement, the samples were removed from the solution, rinsed with distilled water for 1 min, and excess liquid on the surface was gently blotted with absorbent paper. The L*, a*, and b* values were measured three times under the same conditions as the initial measurements, and the averages were recorded.

The color change at each evaluation time point was calculated using the CIEDE2000 formula, with the T0 values of the same sample serving as the reference. Thus, the values ΔE_00_(T0–T1), ΔE_00_(T0–T7), ΔE_00_(T0–T14) and ΔE_00_(T0–T28) were obtained, respectively.

### 2.10. Statistical Analysis

Statistical analyses were performed using IBM SPSS Statistics for Windows, Version 25.0 (IBM Corp., Armonk, NY, USA). The normality of continuous data was assessed using the Shapiro–Wilk test for each subgroup and variable. Data showing a normal distribution were expressed as mean ± standard deviation, while data not showing a normal distribution were expressed as median and interquartile range (Q1–Q3).

For the time-dependent changes in color change (ΔE_00_) values on days 1, 7, 14, and 28, one-way repeated-measures analysis of variance (repeated-measures ANOVA) was used for data following a normal distribution, while the Friedman test was used for data not following a normal distribution. In the repeated-measures ANOVA, the sphericity assumption was assessed using the Mauchly test, and the Greenhouse–Geisser correction was applied when the assumption was violated. Where significant differences were detected, pairwise comparisons were performed using the Bonferroni-corrected paired *t*-test for normally distributed data and the Bonferroni-corrected Wilcoxon signed-rank test for non-normally distributed data.

When comparing water sorption (Wsp) and solubility (Wsl) values across processing times of 3, 10, and 30 min (independent groups), one-way ANOVA was used for data following a normal distribution, and the Kruskal–Wallis test was used for data not following a normal distribution. Where significant differences were detected, pairwise comparisons were performed using the Bonferroni-corrected independent-samples *t*-test for normally distributed data and Dunn’s test with Bonferroni correction for non-normally distributed data.

To directly assess the combined effects of material and processing duration, two-way ANOVA models (material × duration) were additionally fitted for Wsp and Wsl within each experimental arm. For ΔE_00_, a three-way split-plot ANOVA (material × duration × day) was performed within each arm to evaluate the main effects of material, duration, and day, together with their two- and three-way interactions.

When comparing two materials under the same processing time and protocol, the independent-samples *t*-test was used for data following a normal distribution, and the Mann–Whitney U test was used for data not following a normal distribution. When comparing each experimental group to its own control group, the Dunnett test was used for data that followed a normal distribution, and the Mann–Whitney U test with Bonferroni correction for three comparisons was used for data that did not follow a normal distribution. Effect sizes (Cohen’s d, rank-biserial correlation r, or partial eta-squared, as appropriate) were calculated alongside *p*-values in Table 2, Table 3, Table 4, Table 5, Table 6, Table 7, Table 8 and Table 9. All analyses were performed as two-tailed tests, and a *p* < 0.05 level was considered statistically significant.

## 3. Results

### 3.1. Color Stability

**Figure 2:**
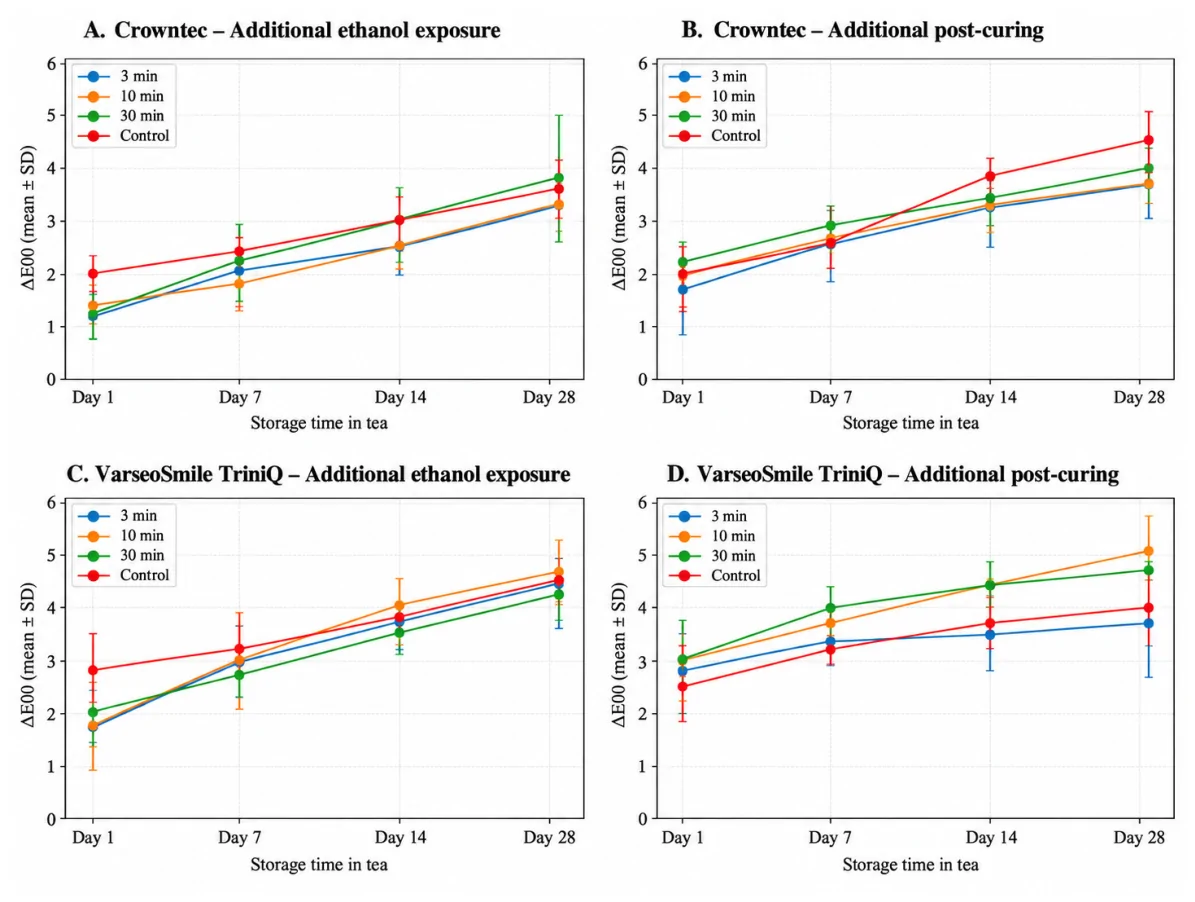
Time-dependent change in ΔE_00_ for Crowntec and VarseoSmile TriniQ according to additional ethanol exposure and post-curing times.

With the exception of the VarseoSmile TriniQ group that received an additional 3-min post-curing, ΔE values changed significantly over time in all groups (*p* < 0.001); in the group that received the additional 3-min post-curing, however, the overall effect of time was not significant (*p* = 0.086). In general, ΔE values increased with exposure time. While no significant differences were observed between early time points in some of Crowntec’s additional ethanol exposure groups, ΔE values increased at later time points. In VarseoSmile TriniQ, however, a significant increase was observed in sequential measurements in the additional ethanol exposure groups and in the 10- and 30-min additional post-curing groups (Figure 2).

When Crowntec and VarseoSmile TriniQ were compared at the same processing times and time points, VarseoSmile TriniQ exhibited higher ΔE_00_ values in all comparisons found to be statistically significant. This difference was particularly pronounced in the 10- and 30-min additional post-curing groups, where VarseoSmile TriniQ’s ΔE_00_ values were significantly higher than those of Crowntec at all time points in both groups. In the additional ethanol exposure groups, however, the difference between the materials varied depending on time and application duration; in the 3- and 10-min groups, the differences were particularly pronounced on days 7, 14, and 28 (Table 2).

When the experimental groups were compared with their respective control groups, all groups exposed to additional ethanol in both materials showed ΔE_00_ values that were significantly lower than those of the control only on Day 1; no significant differences were observed at subsequent time points. The effect of additional post-curing varied depending on the material; for VarseoSmile TriniQ, the 10-min treatment resulted in higher ΔE_00_ values than the control on days 7, 14, and 28, while the 30-min treatment resulted in higher ΔE_00_ values than the control on days 7 and 14. In Crowntec, however, lower ΔE_00_ values than the control were observed only in the 30-min additional post-curing group on days 14 and 28 (Table 3). Clinical interpretation of these findings was additionally based on the predefined perceptibility (PT = 0.8) and acceptability (AT = 1.8) thresholds. All groups exceeded the perceptibility threshold from Day 1 onward, whereas some Day 1 values remained below the acceptability threshold. From Day 7 onward, all groups exceeded the acceptability threshold. Therefore, statistical significance between groups did not necessarily indicate a difference in clinical acceptability when the compared values remained within the same threshold category.

### 3.2. Water Sorption

Following reanalysis using independent-group statistical tests, no significant effect of treatment duration on water sorption was observed in the Crowntec–additional ethanol exposure arm or in the VarseoSmile TriniQ–additional post-curing arm (*p* = 0.990 and *p* = 0.912, respectively). In contrast, Crowntec showed a significant difference among additional post-curing durations (*p* = 0.013); the 30-min group exhibited significantly lower Wsp than both the 3- and 10-min groups, whereas the latter two did not differ significantly from each other. In the VarseoSmile TriniQ–additional ethanol exposure arm, a significant overall difference was also detected among durations (*p* = 0.002); the 30-min group showed significantly higher Wsp than the 10-min group, whereas the 3-min group did not differ significantly from either the 10- or 30-min groups (Table 4).

No significant differences in water sorption were detected between Crowntec and VarseoSmile TriniQ in any of the additional post-curing groups (all *p* > 0.05). In the additional ethanol exposure test, significant differences were observed between the materials in the 10- and 30-min groups and the control group, whereas no difference was detected in the 3-min group (*p* = 0.280). Crowntec exhibited higher water sorption than VarseoSmile TriniQ during the 10-min additional ethanol exposure and in the control group, whereas VarseoSmile TriniQ showed higher values in the 30-min treatment (Table 5).

When each experimental group was compared to its respective control group, no significant differences in water sorption were observed in the Crowntec groups with additional ethanol exposure or in the VarseoSmile TriniQ groups with additional post-curing. In Crowntec, water sorption in the 30-min additional post-curing group was significantly lower than in the control (*p* = 0.036), while in VarseoSmile TriniQ, water sorption in the 30-min additional ethanol exposure group was significantly higher than in the control (*p* = 0.012) (Table 6).

### 3.3. Solubility

Following reanalysis using independent-group tests, no significant differences in Wsl were detected among the 3-, 10-, and 30-min conditions within any experimental arm for either material (all *p* > 0.05; Table 4).

Across the tested conditions, VarseoSmile TriniQ exhibited higher Wsl values than Crowntec in the between-material comparisons (Table 7).

When each experimental group was compared to its respective control group, no statistically significant differences in solubility were observed for Crowntec and VarseoSmile TriniQ in either the additional ethanol exposure or the additional post-curing groups (all *p* > 0.05).

### 3.4. Combined Effects of Material and Processing Duration on Wsp and Wsl

To assess whether the effect of processing duration on water sorption and solubility depended on material type, two-way ANOVA models (material × duration) were fitted separately for each experimental arm (Table 8). For water sorption, a significant material × duration interaction was found in both the ethanol exposure arm (F(3,72) = 4.66, *p* = 0.005) and the post-curing arm (F(3,72) = 3.39, *p* = 0.023), indicating material-dependent effects of processing duration. For solubility, VarseoSmile TriniQ showed markedly higher values than Crowntec in both arms (both *p* < 0.001), with a significant material × duration interaction observed only in the post-curing arm (F(3,72) = 5.13, *p* = 0.003); no such interaction was found for ethanol exposure (*p* = 0.266).

### 3.5. Combined Effects of Material, Processing Duration, and Time on Color Change (ΔE_00_)

To evaluate whether the effects of material, processing duration, and time were interdependent in determining color change (ΔE_00_), a three-way split-plot ANOVA (material × duration × day) was performed separately for each experimental arm (Table 9 and Table 10).

In the ethanol exposure arm, significant main effects of material, duration, and day were observed (all *p* ≤ 0.010), as were the material × day and duration × day interactions (*p* = 0.041 and *p* = 0.016, respectively). However, the material × duration interaction was not significant (F(3,72) = 1.34, *p* = 0.269), nor was the three-way interaction (F(9216) = 1.58, *p* = 0.123), indicating that the effect of ethanol exposure duration on ΔE_00_ did not differ between materials.

In the post-curing arm, significant main effects of material and day were accompanied by a significant material × duration interaction (F(3,72) = 7.23, *p* < 0.001), a significant material × day interaction (F(3,72) = 7.41, *p* < 0.001), and a significant three-way interaction (F(9216) = 2.89, *p* = 0.003), demonstrating that the effect of post-curing duration on color change was both material-dependent and evolved differently over time between the two materials.

Taken together, these results demonstrate a significant material × duration interaction for the post-curing protocol, whereas in the ethanol-exposure protocol, the effect of duration on color change, although significant overall, did not differ between materials. Because each material was processed according to its own manufacturer-specific baseline workflow, the observed between-material differences should not be interpreted as reflecting intrinsic material properties alone.

## 4. Discussion

The effects of post-printing processing varied according to the material, processing method, and evaluation period. ΔE_00_ values generally increased over time, except in the 3-min additional post-curing group of VarseoSmile TriniQ. Processing time affected Wsp in some material–protocol combinations but not others, whereas Wsl was largely unaffected by processing duration despite clear between-material differences. Accordingly, H_01_, H_02_, and H_03_ were partially rejected depending on the outcome and material. These findings indicate that the responses to additional post-processing varied between the two material–workflow combinations. However, because each resin underwent its own manufacturer-specific baseline cleaning and curing procedures, the apparent material dependence may reflect both intrinsic material characteristics and differences in baseline processing.

In studies on color stability, Ünlü et al. (2026) evaluated Saremco Print Crowntec and VarseoSmile Crown Plus in black tea, coffee, and distilled water at 37 °C and measured color changes at 24 h and 14 days using the CIEDE2000 system. The study reported that ΔE_00_ values increased significantly over time in the tea and coffee groups and that black tea, in particular, caused the greatest color change in both materials [22]. Similar studies have shown that the coloring medium, material type, and storage duration have significant effects on color change [23,24]; these findings are consistent with the progressive increase in ΔE_00_ observed in the vast majority of the Crowntec and VarseoSmile TriniQ groups as tea exposure time increased in the present study [22]. The 28-day continuous black tea immersion represents an accelerated staining challenge rather than normal beverage consumption; therefore, ΔE_00_ values should be interpreted as comparative measures of staining susceptibility without direct clinical time equivalence. Differences between the two material–workflow combinations may also partly reflect material composition. Crowntec contains an ethoxylated bisphenol-based methacrylate matrix with silanized dental glass and pyrogenic silica (approximately 30–50 wt% inorganic filler), whereas VarseoSmile TriniQ contains a different methacrylate-based matrix, ceramic filler, and TPO photoinitiator. Differences in resin chemistry, filler characteristics, and filler–matrix interactions may influence water uptake and chromogen adsorption or diffusion, thereby affecting staining susceptibility [22,23,24,25]. However, the exact proprietary formulations and surface-level chemical interactions were not characterized in the present study. Moreover, because only black tea was experimentally evaluated, specific preferential interactions with tea constituents relative to coffee or caffeine cannot be inferred from the present data.

Post-printing washing removes surface residues, unpolymerized resin, and extractable low-molecular-weight components [2,6]. However, Jin et al. (2022) reported that prolonged rinsing, although reducing residual monomers, may loosen the polymer network and impair material properties. Similarly, our findings indicate that increasing the duration of additional 99% ethanol exposure did not provide a linear benefit in terms of optical stability. In both materials, additional ethanol exposure for 3, 10, and 30 min resulted in lower ΔE_00_ values on day 1 compared to the control group; however, it is particularly noteworthy that this advantage completely disappeared by days 7, 14, and 28. Importantly, T0 was established only after desiccation, 7 days of distilled-water storage at 37 °C, and re-desiccation. Therefore, the color findings reflect the staining behavior of previously conditioned specimens rather than freshly processed restorations. This conditioning may have altered hydration, extractable components, surface characteristics, and optical behavior, potentially affecting subsequent staining susceptibility. Accordingly, the study cannot determine the immediate effect of the original post-processing protocols on color stability. The early color response may also be related to greater removal of residual monomers during additional solvent exposure; however, this remains hypothetical because residual monomer content was not measured. In addition, the use of 99% ethanol for the additional immersion, rather than the 96% ethanol used in the standard protocols, means that an effect of solvent concentration cannot be excluded [6]. However, prolonged solvent exposure can go beyond surface cleaning to interact with the polymer matrix, causing changes in surface morphology, hardness, and optical properties [8,26,27]. In particular, Abdulai reported that an extended washing time affected the optical properties of VarseoSmile TriniQ more markedly than those of Crowntec, indicating that the post-washing response is material-specific [8]. In our study, however, the material × duration interaction for ΔE_00_ was not statistically significant in the ethanol exposure arm (Table 9), indicating that the effect of washing duration on color change did not differ significantly between the two materials; nevertheless, TriniQ exhibited higher ΔE_00_ values than Crowntec in all meaningful material comparisons at matched time points. The progressive color increase in Crowntec’s 30-min ethanol group suggests that the initial cleaning benefit of prolonged solvent exposure did not translate into long-term color stability. This is consistent with Nejat et al. (2026), who reported that excessive washing can adversely affect surface roughness and optical properties in some 3D-printed restorative resins [27]. Overall, the findings do not support extending solvent exposure beyond manufacturer-validated protocols. Although additional ethanol exposure was associated with lower ΔE_00_ values on Day 1, this benefit was not sustained. Moreover, in VarseoSmile TriniQ, 30 min of additional ethanol exposure was associated with higher Wsp than the 10-min condition and its control, indicating no consistent advantage of prolonged solvent exposure. Therefore, the additional ethanol conditions investigated here should be interpreted as experimental over-processing challenges rather than as alternative or optimized washing protocols. This distinction is particularly important for Crowntec, for which the manufacturer recommends manual alcohol cleaning while avoiding alcohol immersion.

Abdulai (2026) reported that prolonged isopropanol washing increased Crowntec surface roughness without significantly affecting color change [8]. In the present study, the progressive ΔE_00_ increase in the W + 30 group suggests that prolonged solvent exposure may enhance subsequent staining susceptibility. This is consistent with Batmaz et al. (2025), who found that extended post-washing can alter Crowntec physicochemical properties without additional benefit [9]. This mechanism may be related to the progressive color change observed in the Crowntec W + 30 group; however, neither residual monomer extraction nor changes in polymer-network characteristics were directly measured in the present study [6].

Previous studies have reported that post-curing may advance photopolymerization, increase the degree of conversion, reduce residual monomer content, and promote further cross-linking within 3D-printed resin matrices [3,28,29]. These mechanisms provide a possible framework for interpreting post-curing-related changes; however, neither degree of conversion nor residual monomer content was measured in the present study. Aktug Karademir et al. examined Crowntec and VarseoSmile Crown Plus at post-curing durations of 5, 10, and 30 min; they reported that as the post-curing duration increased, the degree of conversion rose and the ΔE_00_ values decreased [3]. In our study, although color change increased over time in all samples in the additional post-curing groups for Crowntec, the fact that the ΔE_00_ values in the C + 30 group were found to be significantly lower than those in the control group on days 14 and 28 suggests an association between extended post-curing and improved long-term color stability in Crowntec under the conditions evaluated. This finding applies to Crowntec within its own curing workflow and should not be extrapolated quantitatively to VarseoSmile TriniQ, for which the baseline curing cycle was dose-controlled rather than time-controlled. Lee et al. (2022) reported that longer post-curing reduced ΔE_00_ in 3D-printed resin specimens exposed to coffee and red wine [5]. In contrast, Feiten et al. (2026) showed that post-curing effects depend on the protocol, with high-irradiance conditions potentially increasing ΔE_00_ beyond the clinical acceptability threshold [30]. In VarseoSmile TriniQ, ΔE_00_ increased significantly over time in the C + 10 and C + 30 groups, which also showed greater color change than the control at several later time points. This is consistent with previous reports that post-curing effects on color stability depend on both the material and protocol [28,31]. The contrasting response of Crowntec indicates that the relationship between post-curing duration and color stability is not universally linear. VarseoSmile TriniQ contains the TPO photoinitiator [10], and Almeida et al. (2026) showed that high TPO concentrations combined with extended post-curing may increase color change and yellowing in 3D-printed restorative resins [25]. These findings suggest that the optical effects of post-curing depend on both duration and material composition. This was particularly evident in the C + 10 and C + 30 groups, in which VarseoSmile TriniQ showed significantly higher ΔE_00_ values than Crowntec at all time points. Because the manufacturer-validated baseline curing protocols differed, identical additional post-curing durations should not be interpreted as equivalent total curing exposure; between-material differences therefore reflect responses within distinct material-specific workflows. Clinical interpretation of ΔE_00_ was based on both the perceptibility (PT = 0.8) and acceptability (AT = 1.8) thresholds. All groups exceeded PT from Day 1, while all exceeded AT from Day 7 onward. Thus, later statistically significant differences mainly indicate differences in the magnitude of discoloration rather than shifts between clinically acceptable and unacceptable color change. Water sorption reflects the hydrophilicity and hydrolytic stability of resin-based materials, as water uptake can promote matrix plasticization, weaken polymer-chain interactions, and impair mechanical properties over time [32,33]. Alharbi et al. demonstrated that an increase in the post-polymerization time reduces residual monomer elution and water sorption in 3D-printed resin composites, reporting that post-curing conditions can determine the material’s hydrolytic behavior [34]. Luo et al. reported lower water sorption and solubility in post-cured than non-post-cured 3D-printed denture-base resins, whereas Altarazi et al. found no significant differences among 20, 30 and 50-min post-curing durations [32,35]. Crowntec showed significantly lower Wsp after 30 min of additional post-curing than after 3 or 10 min (*p* = 0.013) and than its control (*p* = 0.036), whereas no duration-related differences were observed for VarseoSmile TriniQ. This suggests a material-dependent response to additional post-curing. However, because degree of conversion and residual monomer release were not measured, the lower Wsp in Crowntec cannot be directly attributed to enhanced polymerization or reduced residual monomer content. The absence of a significant Wsp effect in VarseoSmile TriniQ indicates that the influence of post-curing is not uniform across materials, consistent with Izzettinoglu and Eroglu, who reported resin-dependent effects of post-curing duration on water sorption [36]. Furthermore, Mujayridi et al., who directly evaluated Crowntec, interpreted the fact that different post-curing devices did not cause a significant change in the water sorption and solubility of permanent crown resins as an indication that these materials may behave relatively stably under specific post-curing conditions [37]. In the ethanol-exposure arm, Wsp did not differ significantly across durations for Crowntec, whereas VarseoSmile TriniQ showed higher Wsp after 30 min than after 10 min (*p* = 0.002) and than its control (*p* = 0.012). Gravimetric Wsl reflects net mass change after water storage but does not identify released compounds; compound-specific elution requires direct chemical analysis. Recent UHPLC–MS/MS and LC–MS/MS studies quantifying Bisphenol A release illustrate this distinction between gravimetric solubility and chemically specific elution [38,39].

Accordingly, the present Wsl data cannot identify the specific compounds responsible for the observed mass changes and should not be interpreted as a direct measure of residual monomer release. Wsl may be influenced by factors such as degree of conversion, residual monomer content, resin-matrix and filler composition, manufacturing technique, and post-processing conditions [34,36]; however, these factors were not chemically quantified in the present study. Additional ethanol exposure and 3-, 10-, or 30-min post-curing did not significantly affect Wsl in either material, suggesting relative stability within the tested range. This is consistent with Mujayridi et al. (2026), who reported no significant differences in water solubility among permanent crown resins subjected to different post-curing devices [37].

Recent findings suggest that longer post-curing does not consistently reduce Wsl, indicating that greater polymerization does not necessarily produce a linear improvement in solubility [36,40]. Likewise, Batmaz et al. reported a significant washing time × material interaction for water solubility, supporting a formulation-dependent solvent response [9]. The factorial analysis also identified a significant material × duration interaction for Wsl in the post-curing arm (Table 8). Although VarseoSmile TriniQ exhibited higher Wsl values than Crowntec across the tested conditions, this finding should be interpreted as a between-material difference under their respective manufacturer-specific workflows rather than as evidence of intrinsically inferior hydrolytic stability of TriniQ. Differences in resin chemistry and filler content, together with differences in baseline cleaning, baseline curing, and potentially total curing dose, may all have contributed to the observed Wsl differences. Indeed, in Abdulai’s study on hydrolytic stability, a distinct material dependency was observed between the Wsp and Wsl values of different restorative resins, and these differences were attributed to compositional variations in the resin matrix and filler system [16]. Such compositional factors may have contributed to the observed differences, although their effects cannot be separated from those of the manufacturer-specific baseline processing protocols. Negative or near-zero Wsl values, particularly for Crowntec, should therefore be interpreted as apparent negative solubility rather than true negative material loss. These values may arise from incomplete removal of absorbed or bound water during re-desiccation, causing m_3_ to approach or slightly exceed m_1_ [16,40]. Analytical uncertainty should be considered because the balance readability was 0.1 mg; thus, Wsl values based on very small m_1_−m_3_ differences, particularly negative or near-zero values, should be interpreted cautiously. Although additional processing durations did not significantly alter Wsl within either material, factorial analysis showed a significant material × duration interaction in the post-curing arm. The higher Wsl values of VarseoSmile TriniQ therefore represent a between-material difference under the tested workflows and cannot be attributed solely to intrinsic composition. Study strengths include a priori sample-size determination, randomized group allocation, standardized procedures, independent experimental arms, and repeated CIEDE2000-based color assessment alongside Wsp and Wsl measurements. The main limitations include the in vitro design, evaluation of only two 3D-printing resins, and use of a single chromogenic medium (black tea). The 28-day continuous tea immersion represents an accelerated staining challenge and should not be assigned a direct clinical time equivalent. In the ethanol arm, differences in manufacturer-recommended baseline cleaning and the use of 99% ethanol for additional immersion mean that the findings reflect material-specific responses rather than the isolated effect of washing duration. Likewise, baseline post-curing protocols differed between materials, and total radiant exposure could not be directly quantified; therefore, identical additional curing times should not be considered equivalent total curing doses, and between-material Wsl differences cannot be attributed solely to intrinsic hydrolytic stability. Finally, incomplete documentation of desiccation parameters limits reproducibility, and the 0.1-mg balance readability increases uncertainty for very small m_1_−m_3_ differences, particularly for negative or near-zero Wsl values. Since intraoral conditions were not simulated, exposure to artificial saliva and standardized brushing cycles were not applied; therefore, the potential effects of these factors on color stability, water sorption, and solubility could not be assessed. Degree of conversion and residual monomer content/release were not measured; therefore, interpretations involving enhanced polymerization or monomer extraction/elution remain hypothetical. Likewise, because the storage medium was not chemically analyzed, gravimetric Wsl could not identify or quantify released resin-related compounds. Another limitation is the sequential use of the same specimens for Wsp/Wsl and color testing; because T0 was recorded after water storage and re-desiccation, the ΔE_00_ findings reflect previously conditioned rather than freshly processed specimens. Future studies should use separate specimen sets for optical and hydrolytic testing and should also assess degree of conversion, residual monomer release, and surface properties.

## 5. Conclusions

Within the scope of this study, the effects of additional ethanol exposure and post-curing varied between the two material–workflow combinations and according to the property evaluated. Additional ethanol exposure was associated with lower ΔE_00_ values than the respective controls on Day 1, but this difference did not persist at later time points. Because T0 was established only after completion of the Wsp/Wsl conditioning procedure, these findings reflect the staining behavior of previously conditioned specimens and should not be extrapolated to freshly processed restorations. In addition, continuous 28-day black tea immersion represents an accelerated staining challenge rather than a direct clinical time equivalent. Within the Crowntec workflow, 30 min of additional post-curing was associated with lower Wsp than the 3- and 10-min conditions and the corresponding control, whereas in VarseoSmile TriniQ, 30 min of additional ethanol exposure was associated with higher Wsp than the 10-min condition and control. Because baseline curing protocols differed between materials, these findings should not be interpreted as comparisons of equivalent total curing doses. No significant within-material differences in Wsl were observed among the additional processing durations. Although VarseoSmile TriniQ showed higher Wsl than Crowntec, this difference should be interpreted in the context of differences in resin chemistry, filler content, and manufacturer-specific baseline processing. Overall, extending solvent exposure beyond manufacturer-validated protocols did not provide a consistent benefit and, under some conditions, was associated with less favorable outcomes. These findings support adherence to validated manufacturer-specific workflows and indicate that the additional ethanol immersions used for Crowntec should be regarded as experimental off-protocol challenges rather than alternative washing protocols.

## Figures and Tables

**Table 1:** Materials used in the study, their manufacturers, and their compositions.

Material Category	Material	Manufacturer	Composition/Ingredients *
Additively manufactured permanent restorative resin	Saremco Print CROWNTEC	Saremco Dental AG, Rebstein, Switzerland	4,4′-isopropylidenediphenol, ethoxylated and 2-methylprop-2-enoic acid; silanized dental glass; pyrogenic silica; initiators; inorganic fillers (≈0.7 μm, 30–50 wt%)
Additively manufactured permanent restorative resin	VarseoSmile TriniQ	BEGO Bremer Goldschlägerei Wilh. Herbst GmbH & Co. KG, Bremen, Germany	4,4′-isopropylidenediphenol, ethoxylated and methacrylic acid (55–80 wt%); benzeneacetic acid, α-oxo-, methyl ester (<5 wt%); photoinitiator (TPO); ceramic filler

* Composition according to the manufacturers’ product information/safety data sheets.

**Table 2:** *p*-values for the comparison of ΔE_00_ values of Crowntec and VarseoSmile TriniQ materials under the same processing conditions.

Processing Condition	Additional Duration	Day 1	Day 7	Day 14	Day 28
Additional ethanol exposure	3 min	0.099 d = −0.78	**0.006** d = −1.38	**<0.001** d = −2.30	**0.002** d = −1.61
	10 min	0.335 d = −0.45	**0.004** r = 0.68	**<0.001** d = −2.79	**<0.001** d = −2.76
	30 min	**0.017** d = −1.17	0.099 d = −0.78	**0.045** r = 0.62	0.374 d = −0.41
	Control	**0.001** d = −1.76	**0.001** d = −1.85	**0.002** d = −1.65	**<0.001** r = 0.88
Additional post-curing	3 min	**0.009** r = 0.62	**0.022** d = −1.13	0.535 r = 0.28	0.982 r = 0.24
	10 min	**0.008** d = −1.33	**<0.001** d = −2.06	**<0.001** d = −2.07	**<0.001** d = −2.56
	30 min	**<0.001** r = 1.00	**<0.001** d = −3.13	**<0.001** d = −2.86	**0.002** d = −1.70
	Control	**0.026** d = −1.08	**0.009** d = −1.31	0.578 d = 0.25	0.107 d = 0.76

The Crowntec and VarseoSmile TriniQ values at the same treatment duration and time point were compared using an independent-samples *t*-test (*p* < 0.05). Effect sizes are presented in parentheses as Cohen’s d or rank-biserial correlation r.

**Table 3:** *p*-values for comparisons of ΔE_00_ values between each experimental group and the control group.

Material/Processing Condition	Additional Duration	Day 1	Day 7	Day 14	Day 28
**Crowntec–Additional ethanol exposure**	3 min	**<0.001** d = −2.13	0.449 d = −0.64	0.196 d = −0.86	0.690 r = 0.35
	10 min	**0.010** d = −1.77	0.095 d = −1.33	0.259 d = −0.85	0.791 r = 0.46
	30 min	**0.001** d = −1.66	0.909 d = −0.24	>0.99 r = 0.06	0.510 r = 0.02
**Crowntec–Additional post-curing**	3 min	0.920 d = −0.22	0.993 d = 0.09	0.155 d = −0.89	0.051 d = −1.21
	10 min	0.791 d = 0.38	0.954 d = 0.21	0.215 d = −1.03	0.225 d = −1.00
	30 min	0.510 r = 0.31	0.194 d = −0.81	**0.003** d = −1.77	**0.006** d = −1.32
**VarseoSmile TriniQ–Additional ethanol exposure**	3 min	**0.004** d = −1.67	0.697 d = −0.46	0.998 d = −0.06	0.873 d = −0.25
	10 min	**0.006** d = −1.43	0.816 r = −0.14	0.627 d = 0.43	0.999 d = 0.06
	30 min	**0.034** d = −1.40	0.267 d = −1.00	0.582 r = 0.42	0.342 d = −0.79
**VarseoSmile TriniQ–Additional post-curing**	3 min	0.839 r = −0.20	0.710 d = 0.45	0.607 r = 0.20	0.759 r = 0.12
	10 min	0.642 d = 0.52	**0.026** d = 1.43	**0.011** d = 1.51	**0.007** d = 1.85
	30 min	0.428 r = −0.22	**<0.001** d = 1.93	**0.006** d = 1.78	0.053 d = 1.22

Each experimental group was compared with its respective control group using the Dunnett test (*p* < 0.05). Effect sizes are presented in parentheses as Cohen’s d or rank-biserial correlation r.

**Table 4:** Water sorption (Wsp) and solubility (Wsl) values according to additional ethanol exposure and additional post-curing durations.

Material/Processing Condition	3 Min	10 Min	30 Min	Effect Size	*p* Value
**Crowntec–Additional ethanol exposure (Wsp)**	0.0069 ± 0.0030	0.0070 ± 0.0023	0.0071 ± 0.0038	η^2^ = 0.001	0.990 ¥
**Crowntec–Additional post-curing (Wsp)**	0.0075 (0.0047–0.0087) ^b^	0.0070 (0.0062–0.0080) ^b^	0.0030 (0.0012–0.0037) ^a^	ε^2^ = 0.248	**0.013 µ**
**VarseoSmile TriniQ–Additional ethanol exposure (Wsp)**	0.0045 (0.0032–0.0060) ^ab^	0.0020 (0.0012–0.0047) ^a^	0.0119 (0.0097–0.0129) ^b^	ε^2^ = 0.388	**0.002 µ**
**VarseoSmile TriniQ–Additional post-curing (Wsp)**	0.0080 ± 0.0074	0.0093 ± 0.0050	0.0083 ± 0.0083	η^2^ = 0.007	0.912 ¥
**Crowntec–Additional ethanol exposure (Wsl)**	0.0015 (0.0010–0.0030)	0.0020 (0.0012–0.0030)	0.0020 (0.0010–0.0020)	ε^2^ = 0	0.764 µ
**Crowntec–Additional post-curing (Wsl)**	0.0025 (0.0010–0.0134)	−0.0025 (−0.0075–0.0097)	0.0070 (−0.0022–0.0281)	ε^2^ = 0.008	0.329 µ
**VarseoSmile TriniQ–Additional ethanol exposure (Wsl)**	0.1737 (0.1664–0.1873)	0.1592 (0.1523–0.2331)	0.2150 (0.1958–0.2866)	ε^2^ = 0.116	0.076 µ
**VarseoSmile TriniQ–Additional post-curing (Wsl)**	0.1866 (0.1388–0.2630)	0.2020 (0.1886–0.2433)	0.1821 (0.1722–0.1869)	ε^2^ = 0.004	0.347 µ

Data are presented as mean ± standard deviation or median (interquartile range, Q1–Q3), depending on the distribution characteristics. ¥: One-way ANOVA, followed by Bonferroni-corrected pairwise comparisons; µ: Kruskal–Wallis test, followed by Dunn’s test with Bonferroni correction. η^2^: eta-squared (one-way ANOVA); ε^2^: epsilon-squared (Kruskal–Wallis test). Differences between values on the same row that do not share the same superscript letter are statistically significant (*p* < 0.05).

**Table 5:** Comparison of water sorption values for Crowntec and VarseoSmile TriniQ materials under the same treatment conditions.

Processing Condition	Duration	Crowntec	VarseoSmile TriniQ	Effect Size	*p* Value
**Additional ethanol exposure**	3 min	0.0059 (0.0047–0.0092)	0.0045 (0.0029–0.0079)	r = −0.29	0.280 β
	10 min	0.0069 (0.0047–0.0092)	0.0019 (0.0007–0.0057)	r = −0.61	**0.019 β**
	30 min	0.0059 (0.0039–0.0099)	0.0119 (0.0087–0.0134)	d = −1.36	**0.015 β**
	Control	0.0055 (0.0039–0.0097)	0.0019 (0.0007–0.0049)	r = −0.61	**0.019 β**
**Additional post-curing**	3 min	0.0073 ± 0.0043	0.0079 ± 0.0074	d = −0.12	0.800 γ
	10 min	0.0074 ± 0.0023	0.0093 ± 0.0059	d = −0.48	0.292 γ
	30 min	0.0036 ± 0.0041	0.0083 ± 0.0083	r = 0.33	0.134 γ
	Control	0.0109 ± 0.0084	0.0037 ± 0.0071	d = 0.93	0.051 γ

Data are presented as mean ± standard deviation or median (interquartile range, Q1–Q3) based on distribution characteristics. β: Mann–Whitney U test; γ: independent-samples *t*-test (*p* < 0.05). Effect sizes are presented in parentheses as Cohen’s d (γ) or rank-biserial correlation r (β).

**Table 6:** *p*-Values for the comparison of experimental groups with their respective control groups in terms of water sorption.

Material/Processing Condition	3 Min vs. Control	10 Min vs. Control	30 Min vs. Control
**Crowntec–Additional ethanol exposure**	0.999 £ d = 0.06	0.993 £ d = 0.10	0.984 £ d = 0.11
**Crowntec–Additional post-curing**	>0.99 § d = −0.55	>0.99 § d = −0.59	**0.036 §** r = 0.67
**VarseoSmile TriniQ–Additional ethanol exposure**	0.202 § r = −0.49	>0.99 § r = −0.02	**0.012 §** r = −0.77
**VarseoSmile TriniQ–Additional post-curing**	0.399 £ d = 0.59	0.204 £ d = 0.90	0.347 £ d = 0.59

Each experimental group was compared with its corresponding control group for its respective material and treatment arm. £: Dunnett’s test; §: Bonferroni-corrected Mann–Whitney U test for three comparisons (*p* < 0.05). Effect sizes are presented in parentheses as Cohen’s d (£) or rank-biserial correlation r (§).

**Table 7:** Comparison of solubility (Wsl) values between Crowntec and VarseoSmile TriniQ at matched additional processing durations.

Processing Condition	Duration	Crowntec	VarseoSmile TriniQ	Effect Size	*p* Value
**Additional ethanol exposure**	3 min	0.0019 ± 0.0021	0.1759 ± 0.0241	d = −10.15	**<0.001**
	10 min	0.0023 ± 0.0012	0.1915 ± 0.0647	d = −4.14	**<0.001**
	30 min	0.0021 ± 0.0015	0.1841 ± 0.2167	d = −1.19	**0.016**
	Control	−0.0942 ± 0.0989	0.1789 ± 0.0407	d = −3.61	**<0.001**
**Additional post-curing**	3 min	0.0068 ± 0.0107	0.1979 ± 0.0699	d = −3.82	**<0.001**
	10 min	−0.0009 ± 0.0133	0.2118 ± 0.0461	d = −6.27	**<0.001**
	30 min	−0.0084 ± 0.0759	0.1852 ± 0.0423	d = −3.15	**<0.001**
	Control	−0.1143 ± 0.0968	0.1968 ± 0.0296	d = −4.34	**<0.001**

Data are presented as mean ± standard deviation. Crowntec and VarseoSmile TriniQ were compared using an independent-samples *t*-test under the same processing conditions and duration (*p* < 0.05). Effect sizes are presented in parentheses as Cohen’s d.

**Table 8:** Two-way ANOVA results for the effects of material, processing duration, and their interaction on water sorption (Wsp) and water solubility (Wsl).

Variable/Arm	Material F(1,72)	*p*	Duration F(3,72)	*p*	Material × Duration F(3,72)	*p*	Partial η^2^ (Material)	Partial η^2^ (Duration)	Partial η^2^ (M × D)
**Wsp–Additional ethanol exposure**	0.53	0.470	5.30	**0.002**	4.66	**0.005**	0.007	0.181	0.162
**Wsp–Additional post-curing**	0.00	0.9999	0.52	0.670	3.39	**0.023**	0.000	0.021	0.124
**Wsl–Additional ethanol exposure**	105.99	<0.001	1.65	0.186	1.35	0.266	0.595	0.064	0.053
**Wsl–Additional post-curing**	328.67	<0.001	5.62	**0.002**	5.13	**0.003**	0.820	0.190	0.176

Two-way ANOVA (Type II sum of squares) was used to evaluate the main effects of material and processing duration, as well as their interaction, on Wsp and Wsl within each experimental arm. Bold values indicate statistical significance (*p* < 0.05). Partial eta-squared (partial η^2^) values are provided for each effect.

**Table 9:** Split-plot ANOVA results for ΔE_00_ in the additional ethanol exposure arm (material × duration × day).

Effect	df	F	*p*	Partial η^2^
Material	1	77.55	**<0.001**	0.519
Duration	3	4.09	**0.010**	0.146
Material × Duration	3	1.34	0.269	0.053
Day	3	279.78	**<0.001**	0.795
Material × Day	3	2.79	**0.041**	0.037
Duration × Day	9	2.32	**0.016**	0.088
Material × Duration × Day	9	1.58	0.123	0.062

Error(between) df = 72 for Material, Duration, and Material × Duration; Error(within) df = 216 for Day and all interactions involving Day. Bold values indicate statistical significance (*p* < 0.05). Partial eta-squared (partial η^2^) values are provided for each effect.

**Table 10:** Split-plot ANOVA results for ΔE_00_ in the additional post-curing arm (material × duration × day).

Effect	df	F	*p*	Partial η^2^
Material	1	50.42	**<0.001**	0.412
Duration	3	2.37	0.078	0.090
Material × Duration	3	7.23	**<0.001**	0.232
Day	3	271.75	**<0.001**	0.791
Material × Day	3	7.41	**<0.001**	0.093
Duration × Day	9	1.98	**0.043**	0.076
Material × Duration × Day	9	2.89	**0.003**	0.107

Error(between) df = 72 for Material, Duration, and Material × Duration; Error(within) df = 216 for Day and all interactions involving Day. Bold values indicate statistical significance (*p* < 0.05). Partial eta-squared (partial η^2^) values are provided for each effect.

## Data Availability

The original contributions presented in the study are included in the article/Supplementary Material, further inquiries can be directed to the corresponding author.

## Supplementary Materials

The following supporting information can be downloaded at: https://www.mdpi.com/article/10.3390/jfb17100510/s1, Supplementary Table S1: Experimental groups and post-printing treatment protocols.

## Abbreviations

The following abbreviations are used in this manuscript:

3D	Three-dimensional
CT	Crowntec
VT	VarseoSmile TriniQ
DLP	Digital Light Processing
Wsp	Water sorption
Wsl	Water solubility
CIE	Commission Internationale de l’Éclairage
CIEDE2000 (ΔE_00_)	CIEDE2000 color-difference formula
PT	Perceptibility threshold
AT	Acceptability threshold
TPO	Diphenyl(2,4,6-trimethylbenzoyl)phosphine oxide
ISO	International Organization for Standardization
ANOVA	Analysis of variance
SPSS	Statistical Package for the Social Sciences
Q1–Q3	First quartile–third quartile
T0	Baseline measurement
T1, T7, T14, T28	Measurements at Days 1, 7, 14, and 28
