# Supplementary material for: Effects of Additional Ethanol Exposure and Post-Curing Time on Color Stability, Water Sorption, and Solubility of 3D-Printed Permanent Restorative Resins
Source: J Funct Biomater. 2026 Oct 11;17(10):510. doi: 10.3390/jfb17100510 (PMC13706549; doi:10.3390/jfb17100510)
Supplement: Supplementary file 1 [file jfb-17-00510-s001.zip › jfb-4587711-supplementary.pdf]

Supplementary Materials

Effects of Additional Ethanol Exposure and Post-Curing Time on Color Stability, Water Sorption, and Solubility of 3D-Printed Permanent Restorative Resins

Merve Güngör [1,*], Abdurrahman Yalçın [2], Hatice Ortaç [3] and Buket Ayna [1]

Table S1. Experimental groups and post-printing treatment protocols.

Material	Group	Experimental condition
VarseoSmile TriniQ	VT-W0	Manufacturer-recommended standard cleaning + standard post-curing
VarseoSmile TriniQ	VT-W+3	Standard cleaning + 3 min additional ethanol exposure + standard post-curing
VarseoSmile TriniQ	VT-W+10	Standard cleaning + 10 min additional ethanol exposure + standard post-curing
VarseoSmile TriniQ	VT-W+30	Standard cleaning + 30 min additional ethanol exposure + standard post-curing
VarseoSmile TriniQ	VT-C0	Manufacturer-recommended standard cleaning + standard post-curing
VarseoSmile TriniQ	VT-C+3	Standard cleaning + standard post-curing + 3 min additional post-curing
VarseoSmile TriniQ	VT-C+10	Standard cleaning + standard post-curing + 10 min additional post-curing
VarseoSmile TriniQ	VT-C+30	Standard cleaning + standard post-curing + 30 min additional post-curing
Crowntec	CT-W0	Manufacturer-recommended standard cleaning + standard post-curing
Crowntec	CT-W+3	Standard cleaning + 3 min additional ethanol exposure + standard post-curing
Crowntec	CT-W+10	Standard cleaning + 10 min additional ethanol exposure + standard post-curing
Crowntec	CT-W+30	Standard cleaning + 30 min additional ethanol exposure + standard post-curing
Crowntec	CT-C0	Manufacturer-recommended standard cleaning + standard post-curing
Crowntec	CT-C+3	Standard cleaning + standard post-curing + 3 min additional post-curing
Crowntec	CT-C+10	Standard cleaning + standard post-curing + 10 min additional post-curing
Crowntec	CT-C+30	Standard cleaning + standard post-curing + 30 min additional post-curing
